# Codon optimization of the adenoviral fiber negatively impacts structural protein expression and viral fitness

**DOI:** 10.1038/srep27546

**Published:** 2016-06-09

**Authors:** Eneko Villanueva, Maria Martí-Solano, Cristina Fillat

**Affiliations:** 1 Institut d’Investigacions Biomèdiques August Pi i Sunyer (IDIBAPS), Barcelona, Spain; 2 Research Programme on Biomedical Informatics, Department of Experimental and Health Sciences, Pompeu Fabra University, Hospital del Mar Medical Research Institute, Barcelona, Spain; 3 Centro de Investigación Biomédica en Red de Enfermedades Raras (CIBERER), Barcelona, Spain

## Abstract

Codon usage adaptation of lytic viruses to their hosts is determinant for viral fitness. In this work, we analyzed the codon usage of adenoviral proteins by principal component analysis and assessed their codon adaptation to the host. We observed a general clustering of adenoviral proteins according to their function. However, there was a significant variation in the codon preference between the host-interacting fiber protein and the rest of structural late phase proteins, with a non-optimal codon usage of the fiber. To understand the impact of codon bias in the fiber, we optimized the Adenovirus-5 fiber to the codon usage of the hexon structural protein. The optimized fiber displayed increased expression in a non-viral context. However, infection with adenoviruses containing the optimized fiber resulted in decreased expression of the fiber and of wild-type structural proteins. Consequently, this led to a drastic reduction in viral release. The insertion of an exogenous optimized protein as a late gene in the adenovirus with the optimized fiber further interfered with viral fitness. These results highlight the importance of balancing codon usage in viral proteins to adequately exploit cellular resources for efficient infection and open new opportunities to regulate viral fitness for virotherapy and vaccine development.

Adenoviruses are double stranded DNA viruses with a short lytic replicative cycle. These pathogens are able to infect metazoans ranging from amphibians to mammals, including birds and reptiles. Pathologies caused by adenoviral infection can affect organs such as the eye (in the case of keratoconjunctivitis^1^) or the respiratory^2^,^3^ and digestive tracts^4^. However, adenoviruses are also the most widely used vectors for gene therapy and virotherapy against cancer^5^. Therefore, the study of these pathogens is interesting, not only because of their biological relevance, but also to obtain valuable knowledge capable of impacting their therapeutic potential.

During the late phase of their replicative cycle, adenoviruses block the translation of cellular proteins by preventing phosphorylation of the translation initiation factor eIF4G through expression of the viral protein 100-kDa^6^. This prevents binding of the translation initiation factor to the cap region of cellular mRNAs. Meanwhile, the late adenoviral genes, which are all expressed under the same promoter, can still be correctly translated thanks to a shared highly organized loop structure, known as the tripartite leader structure, in their 5′-UTR^7^,^8^. In this way, the virus is able to monopolize cellular resources by extensively and exclusively exploiting the translational machinery of its host.

Apart from this active exploitation of the cellular resources, some other passive mechanisms are currently being proposed. Recent studies suggest that deviations in codon usage could have a previously unknown relevance in translational regulation. Codon optimization has been described as a major determinant of mRNA stability^9^. The codon usage of a set of proteins can generate a particular demand of aminoacylated-tRNAs that would represent a limiting factor for mRNA translation in the cell. Availability of tRNAs is regulated by the cell and the unbalanced expression of particular tRNAs favors the translation of sets of genes with a defined codon usage. In this way, unbalanced tRNA expression can be used as a mechanism to generate particular translation programs^10^,^11^. Furthermore, a balanced codon usage has been described as a strategy to maximize the translation of simultaneously transcribed genes, supporting the concept of an intergenic competition for translational resources at the codon usage level^12^. Considering that viruses use the cellular translational machinery to translate their own mRNAs, they are subjected to a high pressure to adapt to the available pool of tRNAs in the cell. However, some viruses such as HIV or herpesviruses maintain codons in their genes which show a low prevalence in their host cells^13^,^14^. In some viruses, such as papillomaviruses, such codon usage has been related to gene expression at specific cell differentiation states in which particular tRNA pools are expressed^15^,^16^. In the case of hepatitis A viruses, use of minority codons has been related to the slow protein translation required for correct protein folding^17^. Modification of codon usage has been described as a strategy for vaccine production: on the one hand, recoding of viral genomes to favor the use of minority codons has been used for virus attenuation^18^,^19^,^20^, on the other hand, optimization of viral proteins is used to increase their translation in a non-viral context^21^,^22^,^23^. Notably, codon optimization is also being explored to increase the expression of exogenous genes in virotherapy. However, the impact of this strategy on viral fitness remains unclear.

In the present study, we have analyzed the codon usage of different adenoviral proteins to look for potential regulation patterns. We have observed that viral proteins can be grouped according to their codon usage. In particular, structural proteins seem to have a clear bias towards G/C ending codons, which are majority codons in the host cell. Surprisingly, the adenoviral fiber, a key structural component for the generation of virions, seems to follow an opposite behavior in all the analyzed adenoviral serotypes. Interestingly, fiber optimization mimicking the codon usage of the rest of structural proteins resulted in some unexpected outcomes. Although we observed increased fiber expression produced in a non-adenoviral context, in replicative adenoviruses fiber codon optimization disturbed both fiber expression and that of other structural proteins. In turn, this led to a drastic reduction of viral replicative capacity. In addition, introduction of an exogenous optimized protein as a late gene in an engineered virus containing the optimized fiber further interfered with viral fitness. In summary, our work points to the importance of a balanced codon usage in the adenoviral context and to the need to consider this codon balance during the generation of engineered viruses.

## Results

### Codon usage helps to discriminate adenoviral protein function

**Figure 1:**
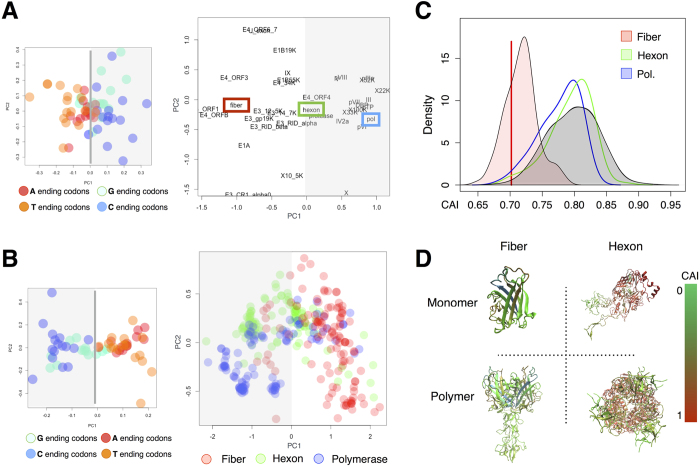
Adenoviral fiber usage of A/T ended codons results in low codon adaptation index to human cells. (**A**) Principal Component analysis (PCA) of all Ad5 proteins: the left panel shows the loadings, which correspond to codons characterized by their usage frequency, the right panel shows the distribution of viral proteins in the two first principal components. The first principal component shows a separation between early regulatory proteins and proteins participating in replication or virion formation. This separation is related to their differential use of A/T ended codons (red and orange) or of G/C ended codons (cyan and blue). Adenoviral fiber, hexon and polymerase are highlighted in red, green and blue respectively. (**B**) Principal Component analysis (PCA) of 117 adenoviral fibers, 103 hexons and 105 polymerases using as loadings codons characterized by their usage frequency as in (**A**). (**C**) Codon Adaptation Index (CAI) analysis according to the human codon usage of all sequenced adenoviral fibers, hexons and polymerases (in red, green and blue respectively) in comparison with human proteins (in grey). The red vertical line corresponds to the CAI of the Ad5 adenoviral fiber. (**D**) Representation of the CAI values along the sequence of the Ad5 fiber and hexon structures in monomeric or trimeric models (with green corresponding to lowest CAI and red corresponding to maximum CAI).

To assess if there was a bias in codon usage that differentiated Ad5 proteins, we initially calculated the frequency of each codon for every Ad5 protein and used these frequencies as protein descriptors in a principal component analysis (PCA). The first principal component discriminated Ad5 proteins according to their function as shown in the PCA scores plot (Fig. 1A). Negative PC1 values characterized most early regulatory proteins, while positive ones corresponded to viral proteins responsible for replication and virion formation such as the polymerase and the virion structural proteins. As previously described for other adenoviruses^24^, analysis of the PCA loadings showed that this protein separation in PC1 could be explained by their differential use of A/T versus G/C at the third codon position (see Figure S1 for a loadings plot specifying all codons and Figure S2 for a representation of correlation between codon CG content and PC1 distribution). Assessment of this separation by a Fisher exact test confirmed its statistical significance (p value < 0.0001).

### Adenoviral fibers have a non-optimal codon usage

Further analysis of the PCA scores showed that the fiber, a late phase structural protein that is crucial for virion assembly, displayed a codon distribution clearly distinct from the rest of structural proteins. To assess whether this is a general trend in adenoviruses, we performed a second PCA with information from more than 100 adenoviral sequences. For this analysis, we selected three proteins with different codon usage in the Ad5: the fiber, the hexon and the polymerase (Fig. 1A, red, green and blue respectively). As shown in Fig. 1B, the first principal component separated the fiber from the other two proteins and this was also related to their differences in the use of A/T versus G/C ended codons (see Figure S3 for a loadings plot specifying all codons).

To analyze how this could affect the way in which these proteins are adapted to the human codon usage we calculated their codon adaptation index (CAI). First, we calculated the codon adaptation index for 700 randomly selected human proteins compared to the mean human codon usage and obtained a mean CAI value of 0.80 (Fig. 1C, gray). The distribution of codon adaptation of adenoviral hexons (green curve) and polymerases (blue curve) from all studied serotypes overlapped with the one of the human proteins. In contrast, adenoviral fibers (pink curve) showed a decrease in CAI greater than the average plus two standard deviations compared to that of the selected human proteins (see Table S1 for a comparison of average codon frequencies per amino acid in fibers, hexons and polymerases). The Ad5 fiber (red vertical line) showed a low level of codon adaptation with a CAI value of 0.70.

To assess whether CAI distribution follows a particular pattern along protein structure, we took advantage of the crystallographic data available for the Ad5 fiber and hexon proteins and we plotted CAI values in their 3D structures (Fig. 1D). The fiber showed lower CAI values along all the structure, as shown by the green trace. In contrast, the hexon displayed core areas in red, indicating high CAI values (Fig. 1D). Notably, CAI distribution in the hexon didn’t show a statistical difference between regions with varying secondary structure such as turns and beta-sheets or strands (t-test, p-value = 0.709, hexon mean CAI values and standard deviation of 0.820 and 0.232 for turn regions and of 0.828 and 0.241 for strands). In the case of the fiber, there was a statistically significant difference between these regions with an increase in CAI in turns as compared to beta-sheets (t-test, p-value = 0.002, fiber mean CAI values and standard deviation of 0.793 and 0.192 for turn regions and of 0.693 and 0.262 for strands). This observation points to the fact that the adenoviral fiber does not seem to require high CAI values in its strand regions for its correct translation as observed in other proteins^25^,^26^.

### Codon optimization of the fiber increases its expression in a non-adenoviral context

**Figure 2:**
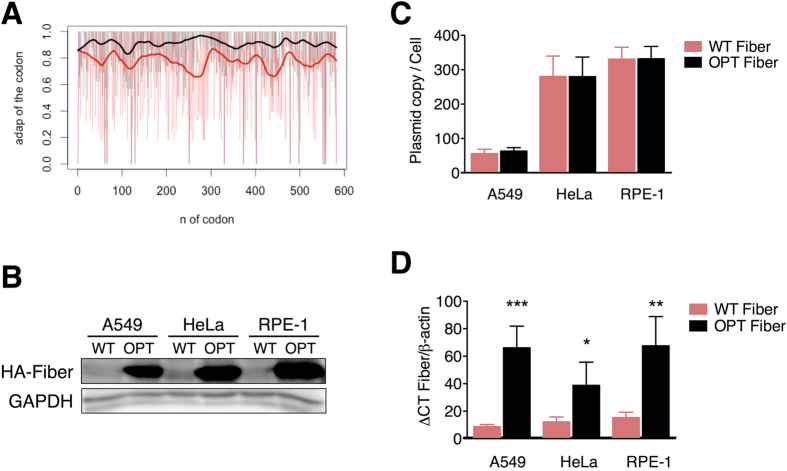
Codon optimization of the fiber increases its expression in transfected cells. (**A**) CAI comparison between the wild-type adenoviral fiber (WT Fiber, red) and the newly engineered codon optimized fiber (OPT Fiber, black). CAI values of each codon are represented raw and smoothened for both proteins. (**B**) Representative western blot of WT and OPT tagged fibers in A549, HeLa and RPE-1 cells at 48 h post transfection. (**C**) Absolute quantification of plasmid DNA content per cell by qPCR. (**D**) qPCR analysis of WT and OPT fiber mRNA levels in the indicated cells. Data is shown as mean ± SEM of four independent experiments. *p < 0.05, ***p < 0.001. Fibers mRNA values are expressed as relative to cellular β-actin in each replicate. *p < 0.05, **p < 0.01.

Codon usage bias is known to have an impact on protein expression^9^. In this line, we decided to recode the Ad5 fiber optimizing its codon usage and evaluate the impact of this codon optimization in protein expression. To this end, we constructed a synthetic fiber with a codon usage that mimics the one of the hexon structural protein (Figure S4). This recoded fiber was considered an optimized fiber for expression in human cells as it showed a higher CAI all along its sequence with respect to the wild-type fiber (Fig. 2A). Taking into account that codon optimization can generate an increase on CpG dinucleotide content in the optimized gene leading to a higher antiviral response, we made sure that our optimization process did not result in a significant increase in CpG dinucleotide content in the adenoviral genome (Fisher exact test, p-value = 0,6435) and that no CpG islands had been formed in the process (Figure S5). In parallel, we assessed if the codon pair bias, a proposed regulator of mRNA translation^27^, had been modified in the process. As can be observed in Figure S6, codon optimization resulted in an optimized fiber using minority codon pairs as compared with the wild-type fiber. To assess the effects of both codon optimization and of the use of minority codon pairs, both fibers were cloned in expression plasmids and transfected into A549, HeLa and RPE-1 cells. Expression analysis at 48 hours post-transfection revealed a remarkable increase in protein content in the cells transfected with the optimized fiber (Fig. 2B). This increase was not related to different DNA levels, as DNA content of both plasmids was equivalent in cells transfected with the wild-type and the optimized fiber (Fig. 2C). Conversely, analysis of mRNA levels showed an increase in all cell lines transfected with the optimized fiber (Fig. 2D). These data highlights the importance of using optimal codons - adapted to the host codon usage - for optimal transgene expression and is consistent with recent observations showing that codon optimality is the main contributor to mRNA stability and that this increase in mRNA stability favors protein expression^9^.

### Fiber codon optimization limits translation of viral structural proteins and compromises viral fitness

**Figure 3:**
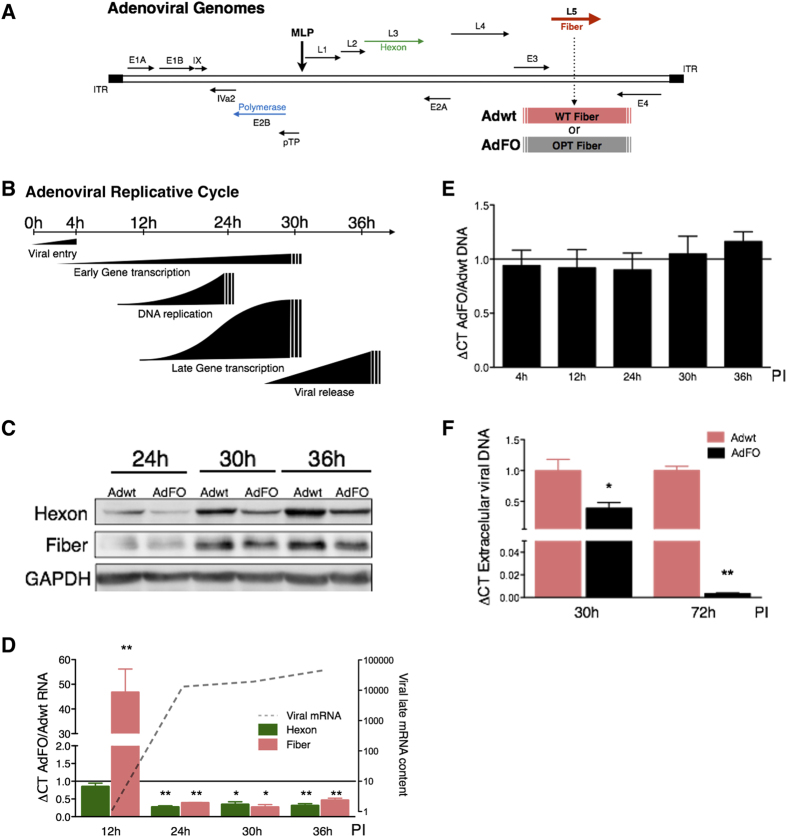
Fiber codon optimization in the adenovirus reduces expression of viral structural proteins and attenuates viral production. (**A**) Schematic representation of wild-type (Adwt) and fiber-optimized (AdFO) adenoviral genomes. (**B**) Graphic description of the time-course of key adenoviral events in infected cells. (**C**) Representative western blot of hexon and fiber protein expression at indicated times. (**D**) Viral mRNA content analyzed at early (12 h), mid (24), and late (30/36 h) phases post infection. Relative AdFO hexon (green) and fiber (pink) mRNA content is shown as mean ± SEM of four independent experiments. Fiber mRNA increase over time is indicated with a grey dotted line. (**E**) Quantification of intracellular viral DNA content by qPCR. (**F**) Extracellular viral DNA release analyzed by qPCR at 30 h and 72 h. Cells were infected using 10 TU/cell of both viruses. Data is shown as mean ± SEM of five independent experiments. All AdFO DNA, mRNA and viral release values are expressed as relative to their corresponding value for Adwt in each replicate. *p < 0.05, **p < 0.01.

To investigate how fiber optimization could affect viral fitness we generated an Ad5 virus, substituting the wild-type fiber for the optimized fiber (named AdFO, Fig. 3A). A549 cells were infected with 10 MOIs of the Adwt and the AdFO virus and fiber protein expression was analyzed at time points of the adenoviral replicative cycle in which late proteins are produced (Fig. 3B). Unexpectedly, AdFO infected cultures showed reduced fiber expression than those infected with Adwt (Fig. 3C). More surprisingly, the hexon and penton wild-type structural proteins also displayed lower expression (Figs 3C and S7). In contrast, expression of the early protein E1A was unaffected showing similar content in AdFO and Adwt infected cultures (Figure S8). To gain insight into the potential causes of the observed reduction of fiber expression, we analyzed the mRNA content of Adwt and AdFO infected cells. At early infection stages, 12 h PI, when mRNA levels of late phase viral proteins are still low, we observed a robust increase in the mRNA content of the optimized fiber (Fig. 3D), in line with our previous observations in cells transfected with this fiber variant (Fig. 2D). This effect was only observed in the fiber mRNA, while the mRNA levels of the structural hexon protein were similar in Adwt and AdFO infected cultures. In contrast, at 24 h PI, both fiber and hexon mRNA levels from AdFO infected cells showed a significant reduction when compared to the levels of Adwt. This behavioral change coincides with a time frame in which there is a massive production of late mRNAs (10.000-fold increase in the mRNA levels of the Adwt fiber, Fig. 3D). As in the case of protein expression (Fig. 3C), the decrease observed in the hexon mRNA was particularly intriguing, since both Adwt and AdFO viruses contain the wild-type hexon gene (Fig. 3A).

Intracellular DNA content at the different time points analyzed was similar in the cultures infected with Adwt or AdFO, discarding that the observed effects were due to differences in viral transduction, genome replication or any other regulatory mechanism previous to viral replication (Figs 3E and S9A). However, despite the intracellular viral DNA content being equivalent in cells infected with both viruses, the alteration in late phase protein expression profile in AdFO infected cells negatively impacted viral production. Thus, quantification of viral release at 30 h PI showed a significant reduction in the presence of viral DNA in the extracellular medium in AdFO infected cultures. These differences were magnified at 72 h PI (Figs 3F and S9B). All together, these observations point to a reduced viral fitness of the AdFO virus. Similar results were confirmed in HeLa and RPE-1 cells, discarding a cell line specific effect (Figure S10A–D).

**Figure 4:**
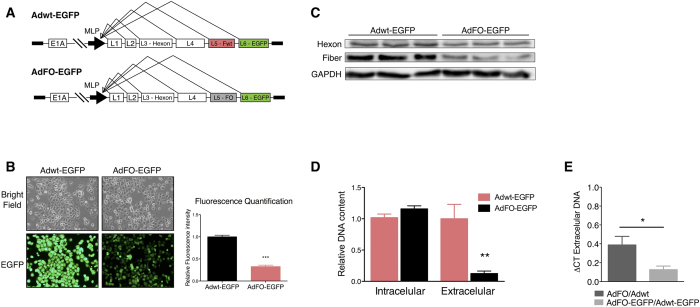
Fiber codon optimization in the adenovirus interferes with the expression of the armed protein when expressed under the control of the Major Late Promoter (MLP). (**A**) Schematic representation of the Adwt-EGFP and AdFO-EGFP genomes with the EGFP engineered as an L6 gene under the control of the MLP. (**B**) EGFP values were analyzed by fluorescence quantification 30 h post-infection in A549 cells infected with Adwt-EGFP or AdFO-EGFP. Left panel shows representative images of EGFP expression. Right panel shows EGFP fluorescence intensity quantification of 20 cells per experiment of three independent experiments shown as a mean ± SEM. (**C**) Representative western blot of hexon and fiber proteins 30 h post-infection. (**D**) Intracellular and extracellular viral DNA content analyzed at 30 h post-infection. (**E**) Comparison of extracellular viral DNA release between AdFO and AdFO-EGFP, versus Adwt and Adwt-EGFP respectively analyzed by qPCR. Cells were infected using 10 TU/cell of both viruses. Data is shown as mean ± SEM of five independent experiments. All AdFO-EGFP fluorescence, intracellular DNA and viral release values are expressed as relative to their corresponding value for Adwt-EGFP in each replicate. ***p < 0.01, ***p < 0.001.

Next, we assessed whether the presence of an optimized fiber would also impact the expression of exogenous proteins when inserted as a late gene under the control of the Major Late Promoter (Fig. 4A). As shown in Fig. 4B, the expression of EGFP was highly reduced in cell cultures infected with AdFO-EGFP when compared to Adwt-EGFP, suggesting that the presence of the optimized fiber was limiting EGFP production. Again, fiber and hexon expression from AdFO-EGFP were significantly diminished with respect to Adwt-EGFP (Fig. 4C). As a consequence, despite no changes were found in the viral DNA intracellular content, a robust reduction in extracellular DNA viral release was detected indicating an attenuation in viral fitness (Fig. 4D). Furthermore, comparison between extracellular DNA viral release in AdFO and AdFO-EGFP infected cells, relative to viruses without the optimized fiber of the same size (Adwt and Adwt-EGFP), showed that introduction of the exogenous EGFP protein leads to an additive effect in terms of loss of viral fitness (Fig. 4E).

## Discussion

Analysis of codon usage by principal component and codon adaptation index analysis revealed that adenoviral proteins could be clustered according to their codon usage. In particular, most early regulatory proteins used codons with A/T endings while proteins implicated in replication and virion formation, including structural proteins, used codons ended in G/C. The adenoviral fiber, however, presented a different codon usage compared to the rest of structural proteins, which was related to a lower adaptation to the host codon usage. In order to assess whether this codon usage had an impact in fiber translation, we engineered a new fiber with an optimized codon usage. As expected, codon optimization of the fiber resulted in a dramatic increase in its expression in transfected human cells. However, infection of human cells with an adenovirus containing the optimized fiber, AdFO, resulted in decreased fiber expression and in a decreased expression of other wild-type structural proteins like the hexon and the penton. This decrease in structural protein expression, in turn, strongly compromised viral fitness. Moreover, introduction of an engineered EGFP gene expressed as a late protein in the AdFO, showed an additive effect on the reduction of viral fitness.

There are several mechanisms that could be associated to the observed results. Codon pair usage has been proposed to affect protein translation and several studies have shown that underrepresented codon pairs are often related to lower translational rates^18^,^27^. In our case, codon optimization of the fiber led to the use of minority codon pairs that could impact its translation, However, despite a reduction in the content of the optimized fiber protein in adenoviral infected cells, individual transfection of the optimized fiber did not lead to a reduction but to an increase in protein expression, precluding a codon pair usage mediated effect. Moreover, this mechanism would not explain the limited translation rates observed for other unmodified structural proteins upon infection with the AdFO virus. Importantly, an alternative explanation could be related to an increase of CpG content upon fiber optimization leading to an increased anti-viral response^28^. In this case, the fact that we did not observe a significant increase of CpG dinucleotides in the AdFO adenoviral genome, nor the formation of islands, leads us to discard this mechanism as the most likely explanation for the observed reduction in protein expression.

Interestingly, there is an alternative mechanism that could help explain the effects of fiber optimization on its translation and on the one of other wild-type structural proteins. As previously mentioned, after an early DNA replication phase, adenoviruses are capable of blocking cellular translation. In this late phase, lytic viruses maximize the production of virions by an exclusive and extensive exploitation of the cellular translational machinery. In the context of massive protein synthesis, aminoacylated-tRNAs (aa-tRNA)s can be a limiting resource. In this scenario, different codon usage in the genes expressed at the adenoviral late phase could help the virus to maximally exploit the entire cellular aa-tRNA pool. Accordingly, alteration of the codon usage of a given protein could generate an imbalance in aa-tRNA usage between structural proteins resulting in a competition for the same aa-tRNA pool. This could help explain our observations, in which the modification of the codon usage of the adenoviral fiber to resemble the one of other structural proteins led to a decreased late protein expression and viral fitness. It would also explain why, the inclusion of an additional optimized protein as a late gene enhanced viral attenuation. Therefore, what would appear to be a deoptimized codon usage in the wild-type adenoviral fiber can be in fact a way to optimize the use of available resources in the host cell.

These observations are in line with recent evidence from eukaryotic unicellular organisms, showing that a balanced use of codons can favor protein expression in a context of high protein demand such as in fast cell growth^12^. In fact, tRNA availability has been described as a potential regulatory mechanism of protein expression in physiological and pathophysiological contexts^10^,^11^,^29^,^30^. From our perspective, the adenoviral model can be good one to study the importance of codon usage balance between proteins, as this model allows studying the use of the cellular machinery by a limited number of proteins under the same promoter at a short timescale.

Finally, the results obtained in this paper point to the importance of considering the availability of cellular resources when exploiting codon bias. These different cellular resources can affect both the expression of the optimized protein and also the expression of the rest of proteins with a similar codon usage. Therefore, further work will be required to assess which is the best strategy to exploit balance in codon usage among adenoviral proteins. This can be determinant in the case of engineered viruses armed as late proteins to favor antitumoral treatment^31^,^32^,^33^,^34^,^35^,^36^. In this case, optimizing the engineered protein could have a negative effect in its own synthesis and on viral fitness, thus impacting viral activity. Interestingly, the disruption of codon usage balance and its consequences in viral fitness could open new opportunities for the production of attenuated viruses. At present, viral attenuation for the production of vaccines usually exploits codon deoptimization^37^,^38^,^39^. However, our results suggest that codon optimization-based strategies considering competition for translational resources can be a novel approach for vaccine engineering.

## Methods

### Codon usage analysis

All adenoviral sequences analyzed are publicly available at the AdenoSeq database from the Institute for Veterinary Medical Research of the Hungarian Academy of Science (http://www.vmri.hu/~harrach/ADENOSEQ.HTM). Codon usage frequencies were analyzed by using the Sequence Manipulation Suite of Bioinformatics.org. We corrected the codon usage of each codon with respect to the rest of the codons coding for the same aminoacid. The relative codon usage frequency was used to perform a PCA analysis using the *pca* function implemented in the R v2.14.1 software. Codon Adaptation Index of each sequence was calculated using the CAIcal prediction server^40^ from http://genomes.urv.es/CAIcal. *Homo sapiens* codon usage extracted from the Codon Usage Database was utilized as the reference value to perform the calculations. The human genes analyzed were selected from the Tissue-specific Gene Expression And Regulation database at the Johns Hopkins University^41^. All calculated codon usage and CAI values are available in Supplementary File 2. In order to analyze CAI distribution along the secondary structure of the fiber and the hexon, secondary structure prediction server STRIDE was used to detect regions corresponding to strands and turns^42^.

### Hexon and fiber 3D representations

3D representations of codon adaptation index distribution in representative viral proteins were obtained as follows: in the case of the hexon protein and the knob region of the viral fiber, we took advantage of the availability of their crystal structures for adenovirus serotype 5 (PDB IDs 3TG7 and 1KNB). Furthermore, in order to enrich the 3D representation of the viral fiber by including additional structural information, we took advantage of the crystallized structure of a portion of the shaft region of adenovirus type 2 (PDB ID 1QIU). This structure, which presents a sequence identity of 59% with the same shaft region of adenovirus type 5, represents a reliable template for homology modeling. Therefore, this structure was used as input to generate a model of the shaft region using MOE software with default conditions and forcefield AMBER12:EHT. The resulting fiber and hexon structures, in combination with codon adaptation index values, were used as input to obtain a graphical representation of codon bias distribution using VMD software^43^.

### Adenoviral Fiber optimization

Fiber-OPT was designed in order to match human codon usage, and by extension adenoviral hexon codon usage. The Fiber-OPT sequence and its alignment with the adenovirus 5 wild-type fiber are available in the Supplementary File 1. The Fiber-OPT gene was synthetically synthetized by GenScript USA Inc. (Piscataway, NJ, USA). Upon codon usage optimization, the possible formation of CpG islands was assessed using the EMBOSS Cpgplot prediction server^44^. In parallel, differences in codon pair bias where analyzed using the protocol described by Coleman *et al.*^27^. Briefly, codon pair scores, corresponding to the natural log of the ratio of the observed over the expected number of occurrences of each codon pair, were calculated to be independent both of amino acid frequency and of codon bias. These scores were subsequently used to calculate the codon pair bias for each gene, corresponding to the arithmetic mean of its codon pair scores.

### Cell lines

Human lung carcinoma A549, cervix adenocarcinoma HeLa, and embryonic kidney epithelial HEK-293 cell lines were obtained from the American Type Culture Collection (ATCC, Manasas, VA), and maintained in Dulbecco’s modified Eagle’s medium supplemented with 10% fetal bovine serum (Gibco BRL, Carlsbad, CA). RPE-1 (hTERT RPE-1) retinal pigmented epithelium cell line was kindly provided by Dr. Raul Mendez (Institute for Research in Biomedicine, Barcelona Spain), and maintained in Dulbecco’s modified Eagle’s medium with Nutrient Mixture F-12, supplemented with 7.5% sodium bicarbonate (Sigma-Aldrich St. Louis, MO, USA) and 10% of bovine serum.

### Expression plasmids generation and transfection

Adenoviral wild-type and codon-optimized fibers were amplified using primer sets 11 and 12 (Table S2) by standard PCR procedure. Amplified DNA was digested with XhoI restriction enzyme and introduced into the pHA1 expression plasmid digested with the same restriction enzyme. Both plasmid constructions were tested by DNA sequencing. Expression plasmids were transfected into A549, HeLa and RPE-1 cells by calcium/phosphate-DNA precipitation method. DNA, RNA and protein extracts were collected forty-eight hours after transfection.

### Adenovirus generation

Ad-wt was obtained from the ATCC (Manasas, VA). The AdFO genome was generated by the following steps: The codon-optimized version of the adenoviral fiber, containing short flanking sequences complementary to wild type adenovirus (purchased from GenScript) was digested using Hpa1 and MfeI restriction enzymes and cloned into the pXK3.1 plasmid, generating the pXK3.1-FO. The pXK3.1 plasmid contains two long arms complementary to the adenoviral genome at both fiber extremes. pAdFO was generated by homologous recombination of the pXK3.1-FO with the genome of the wild-type adenovirus in *E. coli* BJ5183 cells (Stratagene, Wilmington, NC) as described in^45^.

Adwt-EGFP was generated following a protocol adapted from Stanton *et al.*^46^ based on homologous recombination in bacteria using double selection with the rpsLNeo cassette^47^. Briefly, rpsLNeo positive-negative selection cassette was introduced by homologous recombination downstream of the adenoviral fiber sequence. Then, the EGFP gene was amplified using the primer set 8 (Table S2). These primers contain long tails homologous to the adenoviral genome, and the splicing acceptor of the adenoviral fiber upstream of its coding sequence. RpslNeo cassette was replaced by homologous recombination with the EGFP amplicon generating the pAdwt-EGFP. AdFO-EGFP generation was performed using the same recombination system used to generate Adwt-EGFP, but using pAdwt-EGFP as a backbone and substituting the wild-type fiber with the rpslNeo cassette. After this step we substituted the rpslNeo cassette with the codon-optimized fiber by homologous recombination, generating the pAdFO-EGFP. The optimized fiber was amplified with the primer set 9.

pAdFO, pAdwt-EGFP and pAdFO-EGFP were transfected into HEK293 cells to obtain a first round of viral particles. All viruses were propagated in A549 cells and purified by cesium chloride banding. The concentration of viral particles (vp/mL) was determined by means of optical density and infectious particles were determined by the detection of intracellular viral genomes by qPCR 4 h post infection.

### Viral genomes quantification

Viral DNA was obtained from cellular extracts or supernatants using UltraClean BloodSpin DNA Isolation Kit (Mo Bio Laboratories, Carlsbad, CA) according to the manufacturer’s instructions. Adenoviral DNA content was quantified by qPCR using SYBER Green I Master plus mix (Roche Diagnostics, Basel, Switzerland) and the primers set 5 (Table S2). Viral DNA is expressed as relative to the cellular DNA content using the albumin 12 intron primers (primers set 6 in Table S2).

### Viral titration

In order to titrate the viruses, we generated a standard curve for viral and cellular DNA genome numbers. First, DNA from the viral stock was purified by the UltraClean BloodSpin DNA Isolation Kit (MO Bio Laboratories, Carlsbad, CA). The same kit was used to purify the genomic DNA of 10 Million uninfected A549 cells. To determine the total number of DNA molecules per volume of viral and cellular stocks we used the following formula:


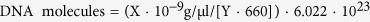


Being X DNA concentration, Y the number of base pairs of the quantified genome, 660 the mean molecular weight of a DNA base and 6.022 · 10^23^ the Avogadro number.

Next, serial two-fold dilutions of the purified viral DNA were performed by triplicate, ranging from 10^7^ to 1 molecules per μl in a background of genomic DNA. For the determination of purified cellular DNA content, serial two-fold dilutions were performed by triplicate, ranging from 100000 to 1 molecules per μl. The absolute number of viral and cellular DNA molecules of each standard dilution was quantified by quantitative real-time PCR. To do so, we used SYBER Green I Master plus mix (Roche Diagnostics, Basel, Switzerland), viral hexon primers set 5 (Table S2) and primers complement to the albumin intron 12 (primers set 6 in Table S2) for cellular DNA quantification. CT values and known DNA concentrations of each viral and cellular dilution were used to generate the standard curves.

In order to titrate the viruses, 400.000 A549 cells were seeded in a 12 multi-well plate. Five serial five-fold dilutions of the different viral stocks were performed (starting at 10^−1^) and 10 μl of each dilution were used to infect the cells, in 3 replicates per virus and dilution. As in all infection experiments, four hours post-infection, medium with non-infective viral particles was removed and cells were washed twice with PBS. Next, DNA from infected cells was purified using UltraClean BloodSpin DNA Isolation Kit (MO Bio Laboratories, Carlsbad, CA) according to the manufacturer’s instructions. Viral and cellular DNA from infection experiments was quantified in a qPCR assay, in parallel to the standards. Standard curves were generated and CT values from infected cells were used to calculate the DNA concentration by extrapolation to the standard curves. This procedure allowed determining the number of DNA molecules of viral and cellular genomes. The ratio between viral and cellular DNA copy numbers resulted in the total number of viral genomes per infected cell.

### cDNA synthesis and qPCR analysis of viral RNA expression

Total RNA was obtained and isolated using RNeasy Mini Kit (Qiagen, Venlo, Netherlands). 0.5 μg of this RNA were reversely transcribed using Moloney Murine Leukemia Virus reverse transcriptase, oligo (dT) and random decamers (Ambion, Carlsbad, CA). Two microliters of the reaction were used as a template for the qPCR amplification reaction (LightCycler 480SYBER Green I Master Mix, Roche) in a ViiA 7 Real-Time PCR thermocycler (Applied Biosystems). Quantitative data was normalized to β-actin expression. All primers used are available in Table S2.

### Western blot analysis

Protein extracts were obtained using a lysis buffer (50 mM Tris-HCl (pH 6.8), 2% SDS) containing 1% Complete Mini Protease Inhibitor (Roche Diagnostics GmbH, Basel, Switzerland). Protein concentration was determined by BCA Protein Assay kit Pierce-Thermo Fisher Scientific, Waltham, MA). In all presented western blots a total of 35 μg of proteins were resolved by electrophoresis on a 7.5% acrylamide gel and transferred to nitrocellulose membranes by standard methods. Membranes were immunoblotted with Adenovirus Fiber [4D2] antibody (1:200; GeneTex, San Antonio, TX) for fiber detection, anti–Adenovirus-2/5 E1A polyclonal antibody (1:200; clone 13 S-5; Santa Cruz Biotechnology, Dallas, TX) for E1A detection, and Anti-Adenovirus Type 5 capsid antibody (1:200; Abcam, Cambridge, UK) for hexon and penton detection. All antibodies were incubated for 1 h at room temperature (RT). Antibody labeling was detected by the enhanced chemiluminescent method (Amersham Biosciences, Amersham, UK). All protein expression data was normalized to GAPDH.

### EGFP quantification

EGFP intensity was quantified by correcting integrated density of the fluorescence intensity in the selected area of each cell using the ImageJ v10.2 software.

### Statistical analyses

Experimental data is represented by the mean ± SEM. Descriptive statistical analysis was performed on GraphPad Prism v5.0a (GraphPad Software, La Jolla, CA). Unless specified, statistical differences were evaluated using a 2-tailed non-parametric Mann-Whitney test. The level of significance was set as p < 0.05. CAI, PCA and CPB analysis are represented using R v2.14.1 software.

## Additional Information

**How to cite this article**: Villanueva, E. *et al.* Codon optimization of the adenoviral fiber negatively impacts structural protein expression and viral fitness. *Sci. Rep.* **6**, 27546; doi: 10.1038/srep27546 (2016).

## Supplementary Material

Supplementary Information
