# Supplementary material for: Codon optimization of the adenoviral fiber negatively impacts structural protein expression and viral fitness
Source: Sci Rep. 2016 Jun 9;6:27546. doi: 10.1038/srep27546 (PMC4899721; doi:10.1038/srep27546)
Supplement: Supplementary Information [file srep27546-s1.pdf]

Supporting Information

Codon optimization of the adenoviral fiber negatively impacts structural protein expression and viral fitness

Eneko Villanueva[1], Maria Martí-Solano[2] and Cristina Fillat[1, 3*]

Table S1. Comparison of average codon frequencies per amino acid in adenoviral fibers, hexons and polymerases.

Statistical significance is analysed using a Kruskal-Wallis test with a Dunns post-test.

aa	Codon	Fiber	Hexon	Polimerase	Fib vs Hex	Fib vs Pol	Hex vs Pol
Ala	GCG	0,10	0,13	0,18	***	***	***
Ala	GCA	0,25	0,13	0,15	***	***	NS
Ala	GCT	0,33	0,26	0,14	***	***	***
Ala	GCC	0,32	0,47	0,54	***	***	*
Cys	TGT	0,58	0,28	0,29	***	***	NS
Cys	TGC	0,42	0,72	0,71	***	***	NS
Asp	GAT	0,51	0,38	0,29	***	***	***
Asp	GAC	0,50	0,62	0,71	***	***	***
Glu	GAG	0,22	0,44	0,57	***	***	***
Glu	GAA	0,78	0,56	0,43	***	***	***
Phe	TTT	0,67	0,42	0,41	***	***	NS
Phe	TTC	0,33	0,58	0,59	***	***	NS
Gly	GGG	0,16	0,14	0,24	NS	***	***
Gly	GGA	0,41	0,23	0,22	***	***	NS
Gly	GGT	0,21	0,24	0,14	*	***	***
Gly	GGC	0,22	0,39	0,40	***	***	NS
His	CAT	0,55	0,26	0,33	***	***	**
His	CAC	0,44	0,74	0,67	***	***	***
Ile	ATA	0,32	0,14	0,14	***	***	NS
Ile	ATT	0,44	0,40	0,17	NS	***	***
Ile	ATC	0,25	0,46	0,70	***	***	***
Lys	AAG	0,28	0,50	0,55	***	***	NS
Lys	AAA	0,72	0,50	0,45	***	***	NS
Leu	TTG	0,13	0,14	0,07	NS	***	***
Leu	TTA	0,21	0,05	0,07	***	***	NS
Leu	CTG	0,13	0,38	0,28	***	***	***
Leu	CTA	0,18	0,07	0,09	***	***	NS
Leu	CTT	0,20	0,14	0,10	***	***	***
Leu	CTC	0,15	0,21	0,40	**	***	***
Asn	AAT	0,49	0,33	0,24	***	***	***
Asn	AAC	0,51	0,67	0,77	***	***	***
Pro	CCG	0,08	0,12	0,19	***	***	***
Pro	CCA	0,32	0,21	0,18	***	***	NS
Pro	CCT	0,25	0,18	0,14	***	***	*
Pro	CCC	0,35	0,49	0,48	***	***	NS
Gln	CAG	0,31	0,65	0,62	***	***	NS
Gln	CAA	0,69	0,35	0,38	***	***	NS
Arg	AGG	0,13	0,13	0,09	NS	**	***
Arg	AGA	0,41	0,25	0,14	***	***	***
Arg	CGG	0,12	0,10	0,12	NS	NS	NS

Arg	CGA	0,10	0,05	0,11	**	***	***
Arg	CGT	0,09	0,08	0,10	NS	**	**
Arg	CGC	0,15	0,39	0,44	***	***	NS
Ser	AGT	0,18	0,13	0,09	***	***	***
Ser	AGC	0,18	0,21	0,22	**	***	NS
Ser	TCG	0,04	0,14	0,14	***	***	NS
Ser	TCA	0,20	0,09	0,10	***	***	NS
Ser	TCT	0,21	0,17	0,13	*	***	***
Ser	TCC	0,20	0,26	0,32	***	***	**
Thr	ACG	0,06	0,14	0,14	***	***	NS
Thr	ACA	0,30	0,18	0,12	***	***	***
Thr	ACT	0,34	0,24	0,15	***	***	***
Thr	ACC	0,30	0,45	0,59	***	***	***
Val	GTG	0,23	0,45	0,34	***	***	***
Val	GTA	0,26	0,13	0,14	***	***	NS
Val	GTT	0,31	0,18	0,12	***	***	***
Val	GTC	0,20	0,23	0,40	NS	***	***
Tyr	TAT	0,53	0,28	0,27	***	***	NS
Tyr	TAC	0,47	0,72	0,73	***	***	NS

Table S2: Primers list

Primer set	Primer name	Primer sequence
1	qPCR-hexon-Fw	GTCTACTTCGTCTTCGTTGTC
	qPCR-hexon-Rv	TGGCTTCCACGTACTTTG
2	qPCR-fiber-Fw	CTCCAACTGTGCCTTTTC
	qPCR-fiber-Rv	GGCTCACAGTGGTTACATT
3	qPCR-fiberOPT-Fw	CTCCCACCGTGCCTTTCC
	qPCR-fiberOPT-Rv	GGCTGACTGTGGTCACATT
4	qPCR-E1A-Fw	CGGCCATTTCTTCGGTAATA
	qPCR-E1A-Rv	CCTCCGGTGATAATGACAAG
5	qPCR-Ad-genome-Fw	GCCGCAGTGGTCTTACATGCACATC
	qPCR-Ad-genome-Rv	CAGCACGCCGCGGATGTCAAAG
6	qPCR-ACTB-Hs-Fw	CTGGAACGGTGAAGGTGACA
	qPCR-ACTB-Hs-Rv	GGGAGAGGACTGGGCCATT
7	qPCR-Albumin-Fw	GCTGTCATCTCTTGTGGGCTGT
	qPCR-Albumin-Rv	GGCTATCCAAACTCATGGGAG
8	RH-Fib-EGFP-Fw	CAATTGGTACTAAGCGGTGATGTTTCTGATCAGCCACCATGGTGAGCAAGGGCGAGG
	RH-Fib-EGFP-Rv	GACTTGAAATTTTCTGCAATTGAAAAATAAAGTTTATTACTTGTACAGCTCGTCCATGC
9	RH-Fib-OP-Fw	GTTCCTGTCCATCCGCACCCACTATCTTCATGTTGTTGCAGATGAAGCGGGCTCGCCCCTC
	RH-Fib-OP-Rv	GTACCAATTGAAAAATAAACACGTTGAAACATAACACAAACGATTCTTTATTCCTGTGCGATATAGCTG
10	Seq-5UTR-Fib-Fw	CAGCTCTGGTATTGCAGCTTCC
11	Fib-WT-ATG-XhoI-Fw	CTGACTCGAGATGAAGCGCGCAAGACCGTCTG
	Fib-Both-XhoI-	CATGCTCGAGGTTTGATTAAGGTACGGTGATCTG

	Rv	
12	Fib-OPT-ATG-XhoI-Fw	CTGACTCGAGATGAAGCGGGCTCGCCCCTCTG
	Fib-Both-XhoI-Rv	CATGCTCGAGGTTTGATTAAGGTACGGTGATCTG

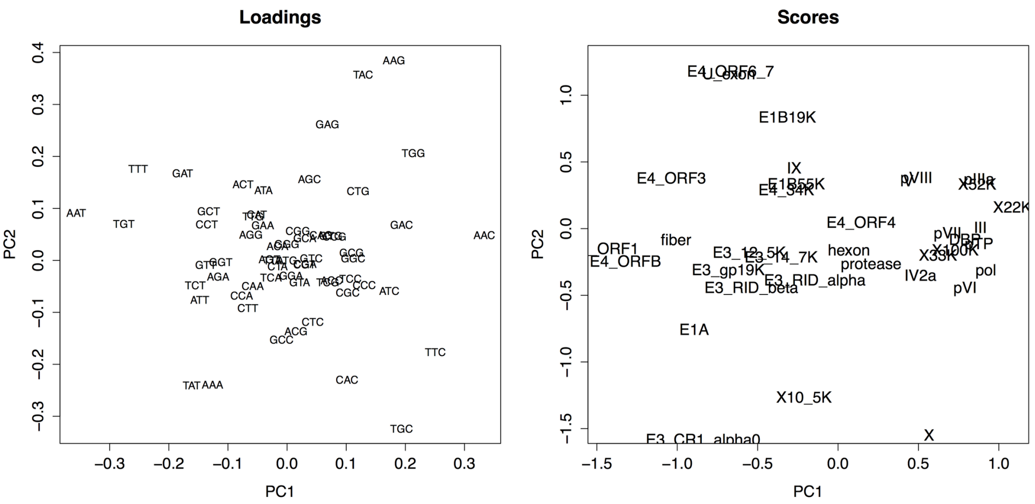

Figure S1. PCA of Ad5 codon usage.

Principal Component analysis (PCA) of all Ad5 genes: the left panel shows the loadings, which correspond to codons characterized by their usage frequency, the right panel shows the distribution of viral proteins in the two first principal components. The first principal component shows a separation between genes coding for early regulatory proteins and genes coding for proteins related to replication and virion formation. This separation is related to their differential use of A/T ended codons or of G/C ended codons.

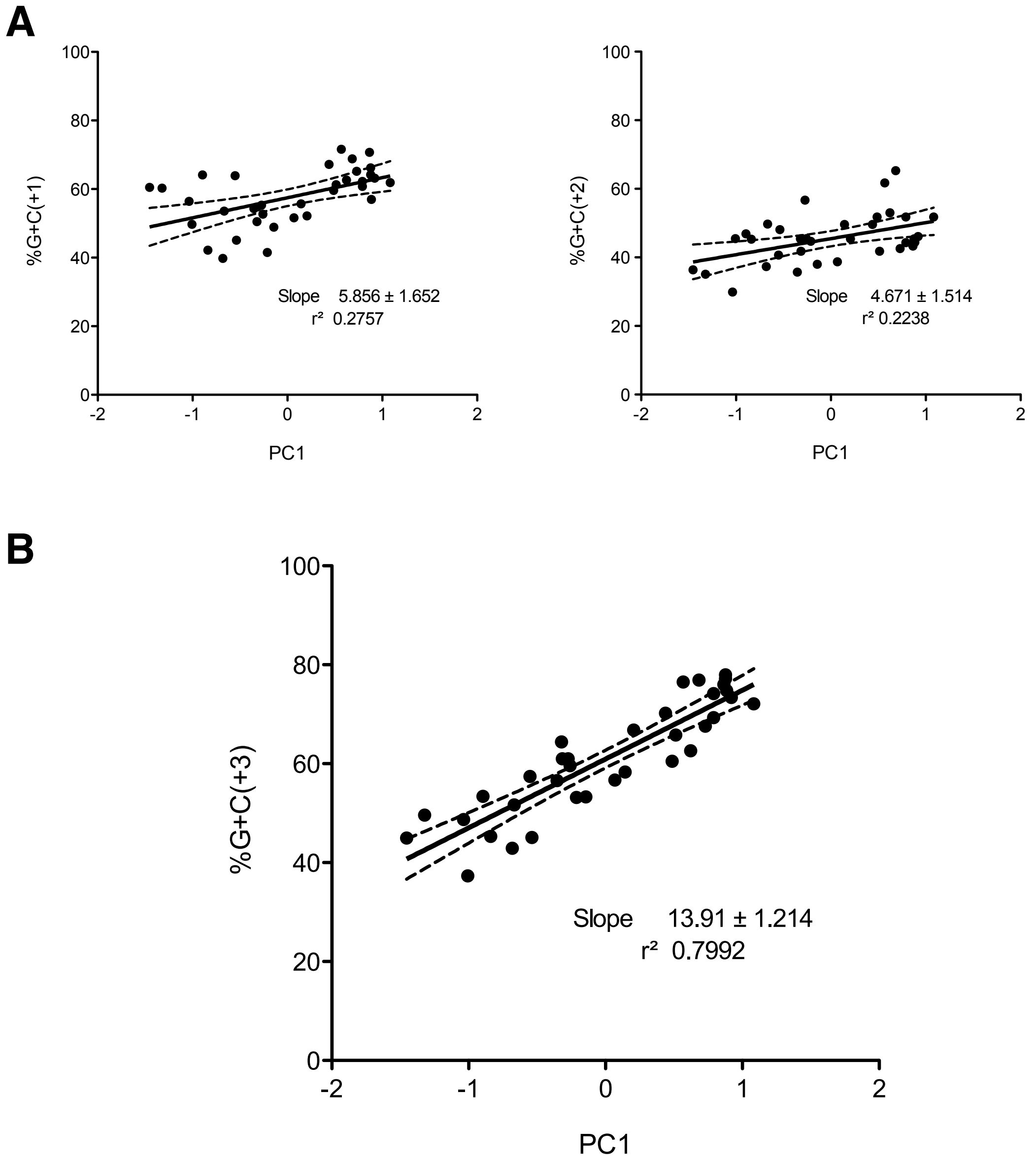

Figure S2. Correlation between codon CG content and PC1 distribution.

(A) C+G values in positions +1 and +2 for each codon of every adenoviral protein do not correlate with the PC1 values.

(B) C+G values in positions +3 for each codon of every adenoviral protein correlate with the PC1 values.

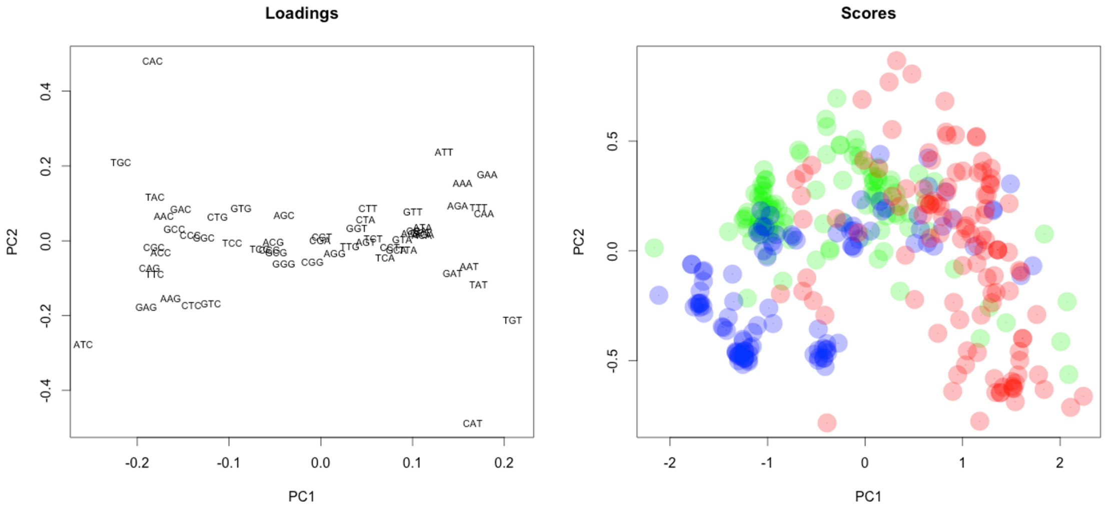

Figure S3. PCA of the codon usage of adenoviral fibers, hexons and polymerases.

Principal Component analysis (PCA) of all sequenced adenoviral fibers (red), hexons (green) and polymerases (blue) using as loadings codons characterized by their usage frequency as in Figure 1A and 1B and Figure S1.

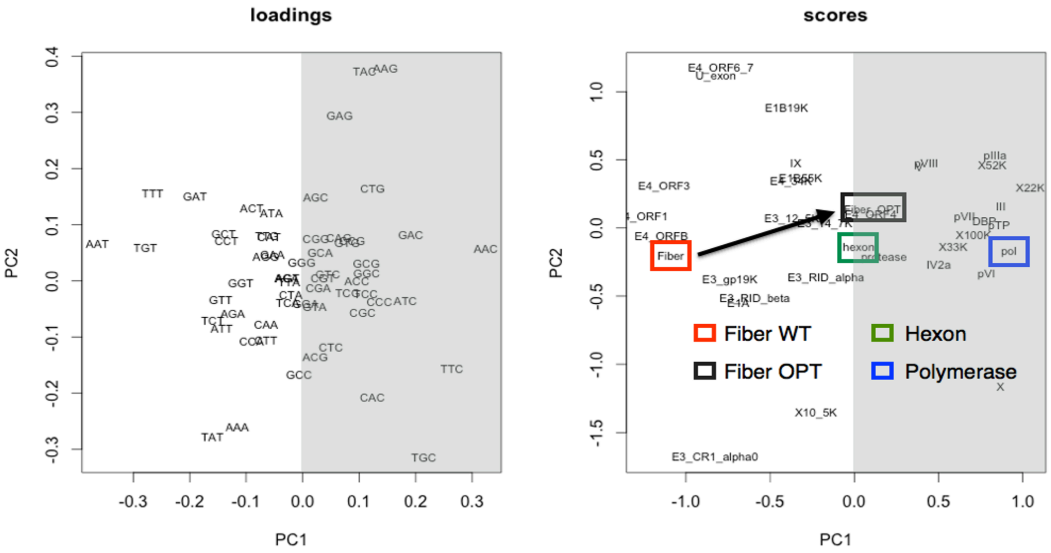

Figure S4. PCA of Ad5 codon usage including the optimized fiber.

(A) Principal Component analysis (PCA) of all Ad5 genes including the optimized fiber (Fiber OPT): the left panel shows the loadings, which correspond to codons characterized by their usage frequency, the right panel shows the distribution of viral genes in the two first principal components and co-localization of the optimized fiber with the genes coding for structural proteins.

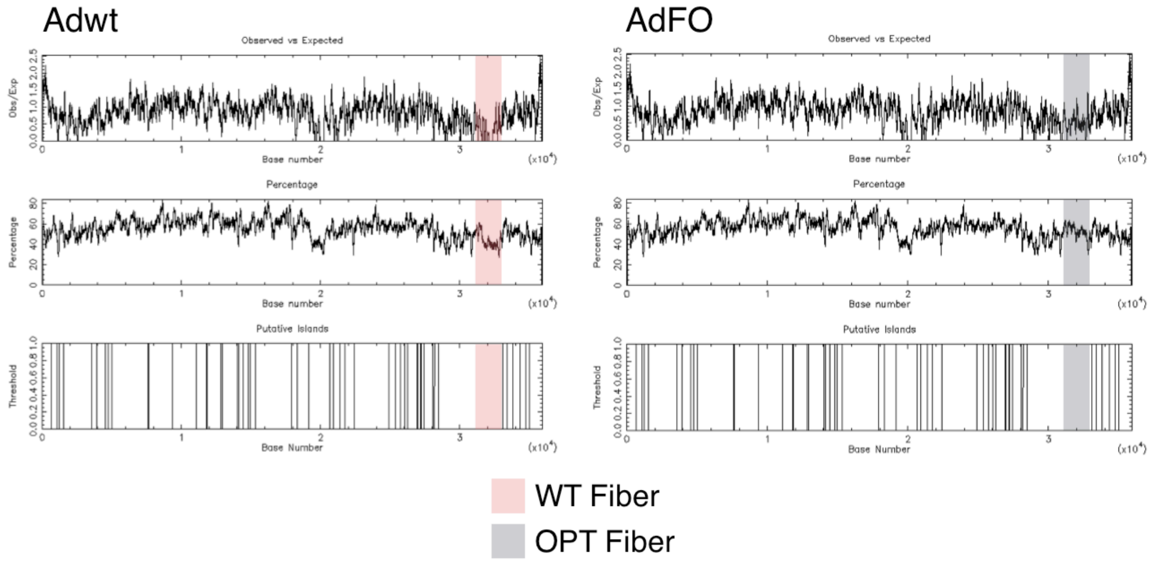

Figure S5. Analysis of CpG islands of Adwt and AdFO genomes.

Up to down: observed versus expected CpG dinucleotide content, percentage of CG, and predicted CpG islands along the adenoviral genome. Coding sequences of adenoviral fibers (WT or OPT fibers) are indicated in pink and grey respectively.

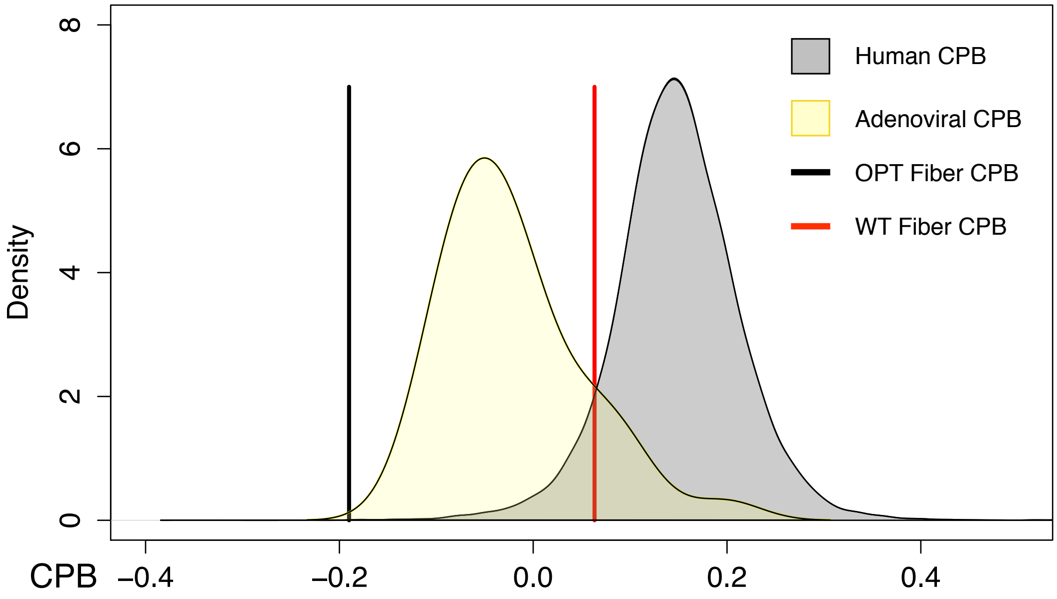

Figure S6. Codon pair bias scores of adenoviral proteins.

Codon Pair Bias (CPB) scores of 14795 human proteins (in grey) and human adenovirus 5 proteins (in yellow), according to the human codon pair usage. The red and black vertical lines correspond to the CPB score of the Ad5 WT fiber and OPT fiber respectively.

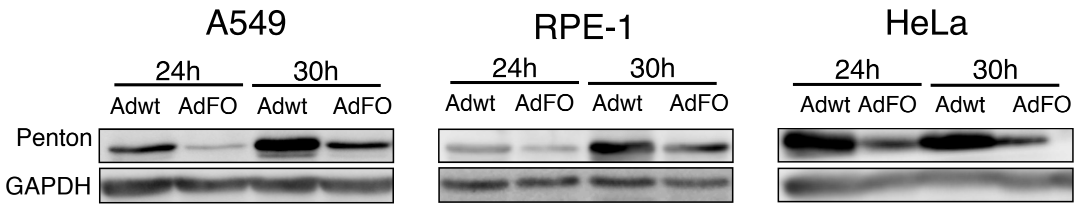

Figure S7. The adenovirus with the optimized fiber (AdFO) expressed reduced levels of the penton protein in A549, RPE-1 and HeLa infected cells. Representative western blot of penton protein expression at two different time points.

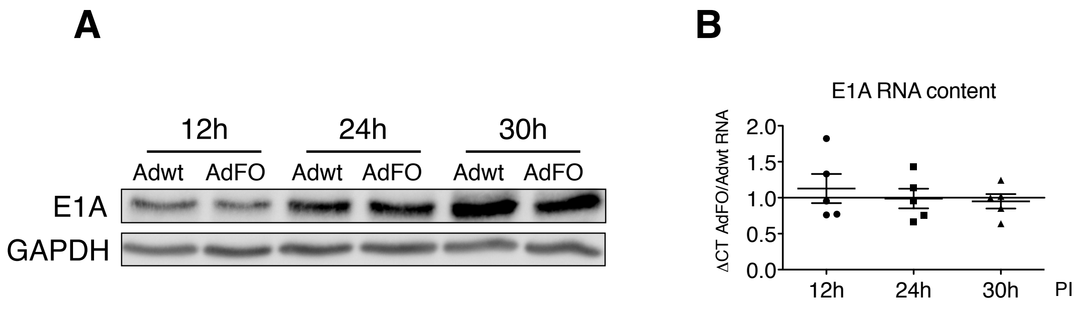

Figure S8. The adenovirus with the optimized fiber (AdFO) displays expression levels of E1A similar to those of adenovirus with the wild type fiber (Adwt).

(A) Representative western blot of E1A protein expression in A549 cultures at indicated time points.

(B) Viral E1A mRNA content analyzed at early (12h), mid (24h) and late (30h) phases post-infection of A549 cultures. Each dot represents an independent experiment.

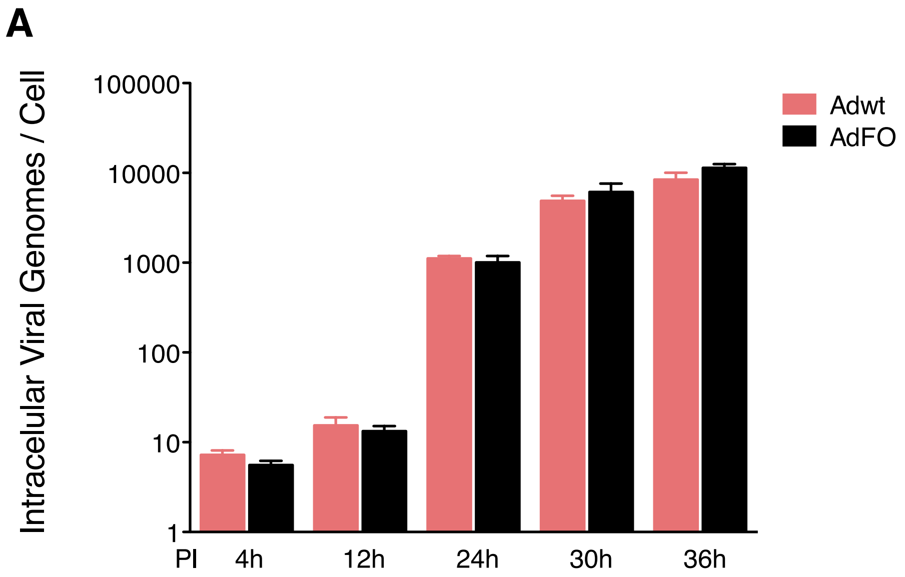

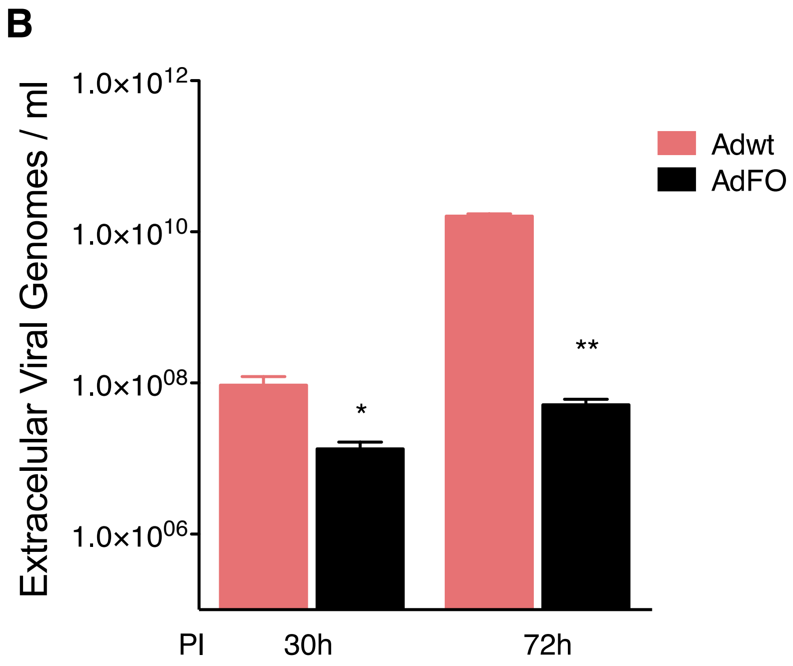

Figure S9. Fiber codon optimization reduces viral production.

(A) Number of intracellular viral genomes per cell determined at the indicated time points.

(B) Number of extracellular viral genomes per milliliter at 30 and 72h.

Cells were infected using 10 TU/cell of both viruses. Analysis of the absolute number of viral genomes was performed by qPCR. Data is shown as a mean ± SEM of five independent experiments. * $p<0.05$, ** $p<0.01$.

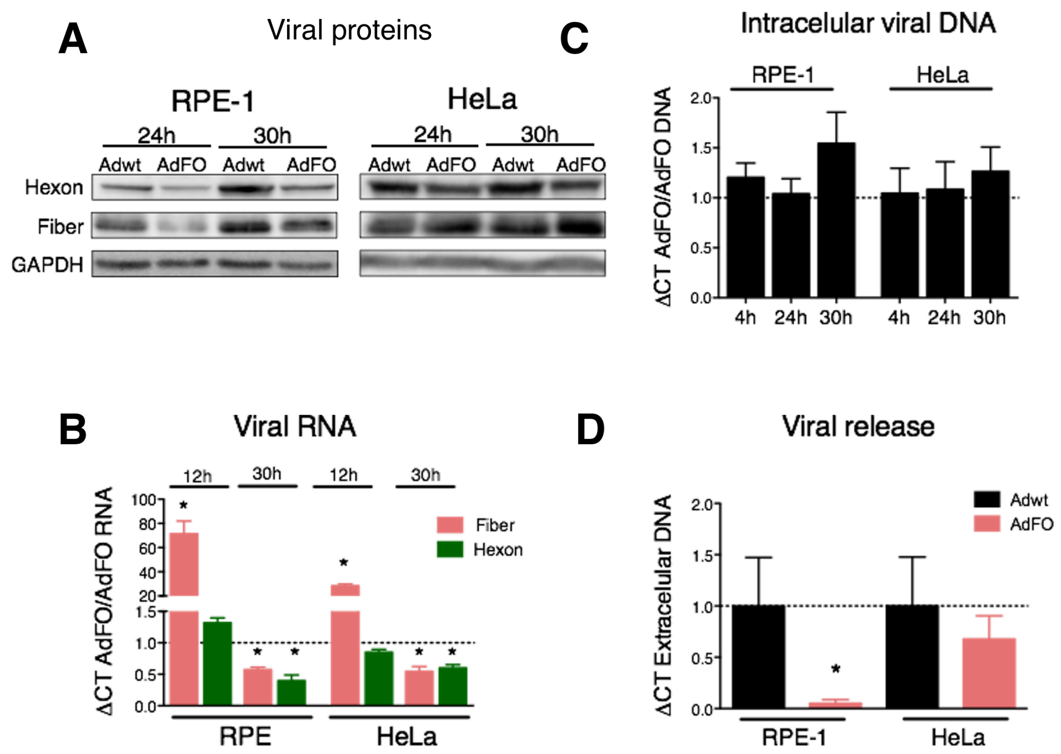

Figure S10. Fiber codon optimization limits translation of viral structural proteins and viral fitness in RPE-1 and HeLa cell lines.

(A) Representative western blot of hexon and fiber protein expression at the indicated time-points.

(B) Viral mRNA content analyzed at early (12h) and late (30h) phases post infection. Hexon and fiber mRNA content is shown as mean ± SEM of four independent experiments.

(C) Quantification of intracellular viral DNA content by qPCR.

(D) Extracellular viral DNA release analyzed by qPCR 30h post-infection.

Data is shown as mean ± SEM of five independent experiments.

All AdFO DNA, mRNA and viral release values are expressed as relative to the corresponding value of Adwt in each replicate. * $p<0.05$.
